# Methylprednisolone Administration Following Spinal Cord Injury Reduces Aquaporin 4 Expression and Exacerbates Edema

**DOI:** 10.1155/2017/4792932

**Published:** 2017-05-10

**Authors:** Eibar Ernesto Cabrera-Aldana, Fernando Ruelas, Cristina Aranda, Ruth Rincon-Heredia, Angelina Martínez-Cruz, Alejandro Reyes-Sánchez, Gabriel Guizar-Sahagún, Luis B. Tovar-y-Romo

**Affiliations:** ^1^Spine Surgery Service, National Rehabilitation Institute, Mexico City, Mexico; ^2^División de Neurociencias, Instituto de Fisiología Celular, Universidad Nacional Autónoma de México, Mexico City, Mexico; ^3^Department of Experimental Surgery, Proyecto Camina A.C., Mexico City, Mexico; ^4^Research Unit for Neurological Diseases, Instituto Mexicano del Seguro Social, Mexico City, Mexico

## Abstract

Spinal cord injury (SCI) is an incapacitating condition that affects motor, sensory, and autonomic functions. Since 1990, the only treatment administered in the acute phase of SCI has been methylprednisolone (MP), a synthetic corticosteroid that has anti-inflammatory effects; however, its efficacy remains controversial. Although MP has been thought to help in the resolution of edema, there are no scientific grounds to support this assertion. Aquaporin 4 (AQP4), the most abundant component of water channels in the CNS, participates in the formation and elimination of edema, but it is not clear whether the modulation of AQP4 expression by MP plays any role in the physiopathology of SCI. We studied the functional expression of AQP4 modulated by MP following SCI in an experimental model in rats along with the associated changes in the permeability of the blood-spinal cord barrier. We analyzed these effects in male and female rats and found that SCI increased AQP4 expression in the spinal cord white matter and that MP diminished such increase to baseline levels. Moreover, MP increased the extravasation of plasma components after SCI and enhanced tissue swelling and edema. Our results lend scientific support to the increasing motion to avoid MP treatment after SCI.

## 1. Introduction

Traumatic injury of the spinal cord generates a lesion that causes severe neurological alterations that afflict thousands of individuals every year, although the exact number of affected people is difficult to assess, especially in low-middle income countries where records are not completely available [1]. There is a limited understanding of the cellular and molecular events that are involved in this pathology and of the processes that contribute to tissue damage and failure of neuroregenerative mechanisms; therefore, therapeutic strategies to treat SCI are scant. To date, there is no pharmacological treatment available for SCI with proven efficacy; the only available protocol currently employed is high doses of methylprednisolone (MP), but its use is highly controversial because the beneficial effects have not been reproducible and are outweighed by severe side effects [2, 3]. More than 30 years ago, MP was considered to reduce lipid peroxidation triggered as secondary damage following SCI [4] and its use is largely justified on the National Acute Spinal Cord Injury Studies trial II (NASCIS II) [5, 6], in which the major finding was that a subgroup of patients treated with 30 mg/kg bolus at hospital admission followed by 5.4 mg/kg/h for the next 23 h starting before 8 h of contusion showed a slight improvement in light touch and pinprick sensation and a very subtle motor improvement. Importantly, the studies that had the potential to validate the NASCIS II trial lack methodological soundness, which limits the ability to draw any conclusions; thus, the positive findings of NASCIS II have never been confirmed with well-designed randomized control trials [7]. Nonetheless, administration of MP following SCI continues to be a common practice [8–11] notwithstanding that it is no longer recommended in the guidelines for the management of acute SCI of the American Association of Neurological surgeons [2].

SCI can be generated by different mechanisms among which are contusions, massive compression, section, and laceration. As a consequence of the primary damage, a series of cellular factors that affect the nearby tissue are released producing inflammation, which in turn increases neuronal damage and the progressive loss of axons, causing secondary damage. Right after the primary lesion, an active state of proinflammatory responses accompanied by tissue swelling and the formation of vasogenic edema takes place [12]. Edema is found by magnetic resonance imaging (MRI) as high signal intensity on T2-weighted and fluid-attenuated inversion recovery images around the injury [13, 14]. It seems that the gray matter is more resistant to water influx than the white matter where fluid accumulation occurs first, which explains why vasogenic edema fluid is just found in the white matter when scanned by computed tomography or MRI [15].

Vasogenic edema is the consequence of an increased permeability of the capillaries that surround the injured area due in part to alterations in the expression of water channels constituted primarily by aquaporins [16] and the dysregulation of permeability factors such as vascular endothelial growth factor [17]. Aquaporin 4 (AQP4) is a member of a water channel protein family expressed primordially in the end feet of astrocytes at the border with the capillary network, the glia limitans and central canal ependyma [18], and the regulation of its expression might underlie some mechanistic actions of MP administration after SCI.

To date, no study addressing the mechanistic effect of MP on spinal edema through the modulation of water channel expression after SCI has been reported. Here, we set out to experimentally determine whether MP administered immediately after contusion contributes to reducing edema and blood-spinal cord barrier (BSCB) alterations following SCI.

## 2. Material and Methods

### 2.1. Animals

Adult male (270–350 g) and female (250–320 g) Long-Evans rats were subjected to severe spinal cord contusion. After the lesion, animals were housed in individual cages in a 12 h light/dark cycle with food and water ad libitum. All animals were killed at 24 h during the acute phase postinjury. For histological analyses, rats were sedated with sodium pentobarbital and subjected to transcardial perfusion with ice-cold 0.9% *w*/*v* NaCl followed by ice-cold 4% *w*/*v* paraformaldehyde. A separate group of animals subjected to the same experimental procedures were killed by pentobarbital overdose, and fresh spinal tissues were collected for edema and Evans blue extravasation quantifications. Experiments were conducted under the guidelines for use and care of laboratory animals (NOM-062-ZOO-1999 Mexico) with approval of the animal care committees of IFC (CICUAL-IFC-LT02-2014) and Proyecto Camina. Every effort was made to minimize animal suffering and number of rats used in this study.

### 2.2. Experimental SCI

Animals were anesthetized with an i.m. mixture of ketamine/xylazine (80/8 mg/kg). Under aseptic conditions, a complete laminectomy was performed at T9. After exposing the dorsal surface of the dural sac and carefully avoiding any dural tear, rats were suspended in the stereotactic frame by clamping T8 and T10 spinous processes. Spinal cord contusion was then produced using the New York University weight-drop device by dropping onto the exposed dura a 10 g rod from a 50 mm height, resulting in an injury of severe intensity [19]. After the lesion, the wound was sutured in layers. Sham-injured animals were only subjected to surgery of soft tissues skipping laminectomy and SCI. To prevent pain and self-mutilation, acetaminophen (Pisa, Guadalajara, Mexico) was given orally at a dose of 30 mg/kg every 12 h. For neurogenic bladder care, manual clenching of the hypogastric region was performed twice over the 24 h period before killing.

### 2.3. MP Administration

In the corresponding groups, rats received an i.p. administration of 30 mg/kg sodium succinate MP (Pisa, Guadalajara, Mexico) 10 min after spinal contusion [20]. Control animals received a corresponding volume of phosphate buffer.

### 2.4. Immunohistochemistry and Confocal Microscopy

Fixed spinal tissues were cut into 40 *μ*m thick transverse sections with a cryostat. Sections adjacent to the site of the lesion were blocked with 5% bovine serum albumin in Tris-buffered saline with 0.1% *v*/*v* Triton X-100 (TBS-T) and incubated with anti-AQP4 (1 : 50; Genetex, Irvine, CA) and anti-GFAP antibodies (1 : 2000; Sigma, St. Louis, MO) for 24 h. Sections were washed 3 times with TBS followed by 2 h incubation at room temperature with Alexa Fluor 488-conjugated anti-mouse and Alexa 546-conjugated anti-rabbit antibodies (1 : 2000 each; Invitrogen, Carlsbad, CA) in TBS. Images were obtained by confocal microscopy (Leica TCS SP5) using a 63x objective. An average of 60 optical slices was obtained every 0.2 *μ*m for each Z-stack. Fluorescence intensity quantification was performed with the image processing package Fiji for ImageJ (NIH) analyzing 8-bit grayscale photograms of images calibrated by area; four images per region were analyzed and averaged for each group. Quantitative data is expressed as arbitrary units of fluorescence.

### 2.5. Evans Blue Extravasation Analyses

Twenty-four hours after SCI, animals received an i.v. bolus of 80 mg/kg Evans blue (2% *w*/*v* in isotonic saline) and after 30 min were sedated with pentobarbital and transcardially perfused with ice-cold 0.9% *w*/*v* NaCl to remove all circulating traces of Evans blue. Fresh tissues were collected and macroscopic photographs were obtained for each specimen; then, tissues were cryopreserved in 30% *w*/*v* sucrose until processed. Injured tissues were homogenized in water, and an equal volume of trichloroacetic acid (60% *v*/*v*) was added to each sample. After a 30 min centrifugation at 10,000 ×g, supernatants were collected and diluted to 1 : 3 in ethanol. A volume of 100 *μ*l was transferred into a black 96-well plate with clear bottom, and Evans blue fluorescence was measured in a microplate reader (BioTek, Winooski, VT) with a 620 nm excitation and 680 nm emission. Relative fluorescence units were converted to concentration by fitting to a known concentration curve and were pondered by protein content to control sample size variability.

### 2.6. Tissue Water Content Determination

Fresh tissues from nonperfused rats were collected, and a segment of 1 cm of the spinal cord containing the lesion was weighed in an analytical scale. Afterwards, tissues were placed on a hot plate (75°C) for 4 h and were weighed again. Tissue water content was calculated as follows:

(1) $$\begin{array}{c} WC=\frac{m_{wet}-m_{dry}}{m_{wet}}\times 100, \end{array}$$

where WC is water content (in percentage of the mass), *m*_wet_ is the mass of the fresh wet tissue, and *m*_dry_ is the mass after tissue was desiccated.

### 2.7. Statistical Analyses

For immunofluorescence, Evans blue, and water content determinations, analyses of variance with Fisher's post hoc tests were performed. A *p*value < 0.05 was considered statistically significant.

## 3. Results

### 3.1. Animals Receiving MP Following SCI Develop Injuries and Hemorrhage

**Figure 1:**
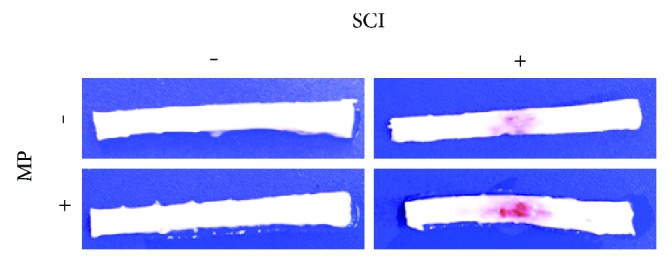
Tissue damage and subarachnoid hemorrhage following SCI. Representative photographs (*n* = 5 per group) of spinal cord segments T5-6 to L1-2 showing the lesion caused by SCI 24 h after contusion. Injured spinal tissues present swelling and subarachnoid hemorrhage in the animals treated with MP and control vehicle.

We analyzed the effect of administering 30 mg/kg MP i.p. 10 min after injury on male and female rats. At 24 h following contusion, the macroscopic lesion observed in the spinal cord was appreciably similar in the animals administered with MP in comparison with injured animals that received a corresponding volume of control vehicle (Figure 1). Subarachnoid hemorrhage also developed in both groups (Figure 1). All animals subjected to SCI presented a full paralysis of the hindlimbs and the tail. According to a locomotor rating scale previously reported [21], there was no appreciable improvement of the motor outcome following MP treatment at this time point, nor were there appreciable differences between injured male and female rats that received MP.

### 3.2. Blood-Borne Molecules Extravasate More Profusely after MP Treatment Following SCI

**Figure 2:**
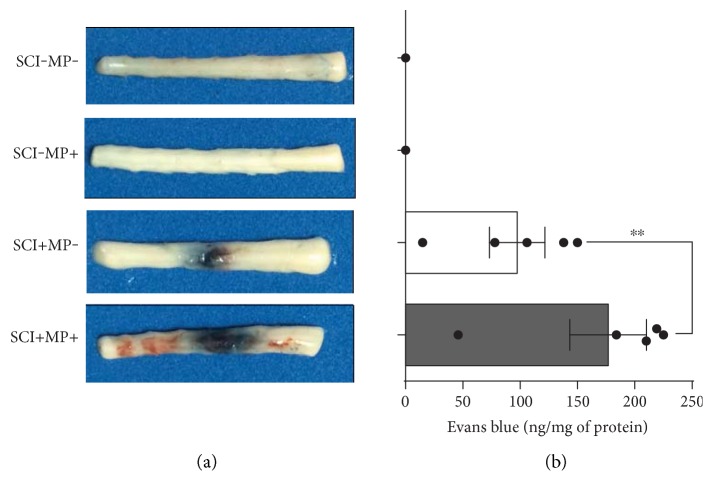
Evans blue extravasation is increased in rats subjected to SCI and treated with MP. (a) Representative photographs of spinal cord segments T5-6 to L1-2 showing the lesion caused by SCI 24 h after contusion and the extravasated Evans blue (*n* = 5 per group). Sham-operated animals do not have any trace of Evans blue in the spinal cord parenchyma; however, the accumulation of this tracer at the site of injury is evident in animals subjected to SCI. MP worsen BSCB disruption causing a further accumulation of the dye at the site of injury. (b) Fluorescence quantification of the extravasated Evans blue shows a notable increase of ~80% parenchymal Evans blue in animals treated with MP related to injured rats administered with vehicle alone. Bars are the mean ± SEM of 5 spinal cord segments; dots show individual data measurements. ^∗∗^*p* < 0.01.

A prominent feature of the SCI at short time points following injury is the disruption of the BSCB, which is considered to increase secondary damage to the spinal tissue [22]. Given the anti-inflammatory properties of MP, it could be hypothesized that the high doses of this corticosteroid may decrease the extravasation of plasma molecules into the spinal parenchyma. We therefore sought to assess whether MP could reduce the extravasation of blood components by i.v. administering Evans blue that noncovalently binds to albumin, the most abundant protein component of blood [23] and measuring its presence in the spinal cord parenchyma at 24 h postinjury. The macroscopic observation of the injured spinal cord revealed a clear increase in extravasated Evans blue stain in the MP-treated animals as compared to animals that received vehicle (Figure 2(a)). Fluorescence quantitative analyses show that there is an ~80% increase in the Evans blue extravasation following MP administration as compared to that in animals that received vehicle, indicating a more impaired state of the BSCB (Figure 2(b)).

### 3.3. Edema Is Higher in MP-Treated Rats Subjected to SCI

**Figure 3:**
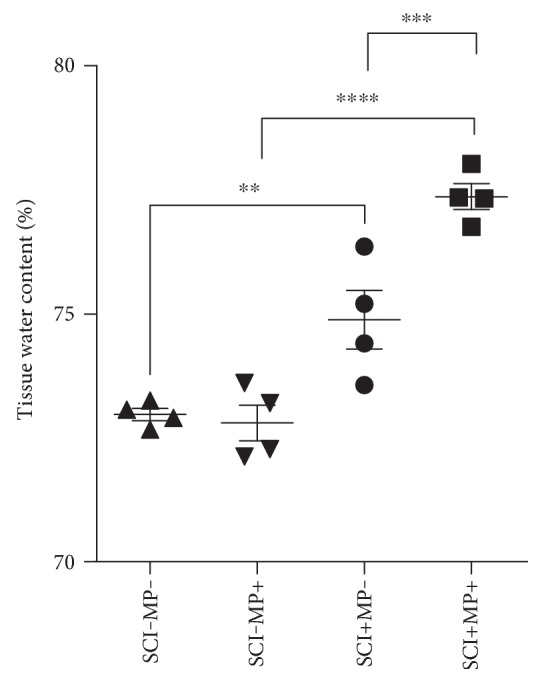
Spinal edema is increased following MP administration. Water content of spinal cord tissues was calculated in 1 cm long segment of the spinal cord at the site of injury. Graph shows statistical mean ± SEM. Each point in the graph shows the percentage of water per spinal cord (*n* = 4 per group). ^∗∗^*p* < 0.01; ^∗∗∗^*p* < 0.001; ^∗∗∗∗^*p* < 0.0001.

The increased extravasation of blood constituents and the swelled volume of the spinal cord at the site of injury in animals treated with MP suggested an increased vasogenic edema. We analyzed tissue water content of the injured segment and the corresponding spinal cord sections from sham-operated rats in order to determine whether MP modified edema formation. We found a statistically significant increase in water content in rats that received MP in comparison with sham-operated rats with and without MP and injured animals that received vehicle only (Figure 3). Injured animals without MP also had a significant elevation of water content as compared to the corresponding group without a lesion and the group of animals treated with MP but without a lesion. MP by itself did not change water content in the spinal cord.

### 3.4. MP Blocks the SCI-Induced Elevation of AQP4

**Figure 4:**
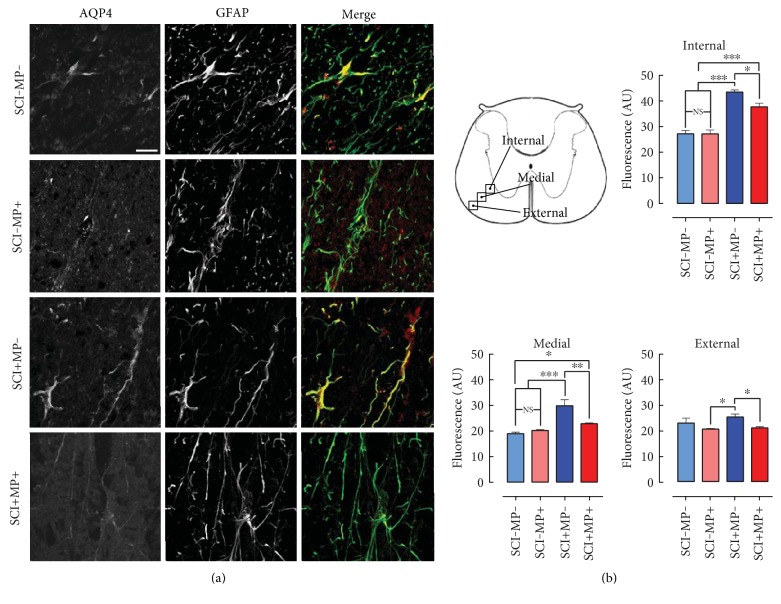
Systemic administration of MP reduces the increased expression of AQP4 in the spinal glia limitans after SCI. (a) Representative micrographs of the white matter glia limitans in the medial zone—indicated in (b)—of spinal cord sections immunostained for AQP4 (red) and GFAP (green) (*n* = 5 per group). Merged signal of AQP4 expressed in astrocytes is depicted in yellow. Bar equals to 20 *μ*m. (b) Quantification of AQP4 immunofluorescent signal in 3 arbitrarily defined regions of the white matter as indicated in the top panel diagram. Values are the mean ± SEM of 4 different photos per region per rat. ^∗^*p* < 0.05; ^∗∗^*p* < 0.01; ^∗∗∗^*p* < 0.001.

Edema formation and resolution are modulated by water channels that mobilize water in and out of cells and tissues. The most abundantly expressed water channel in the CNS is AQP4 that is predominantly expressed in astrocytes [24]; therefore, we sought to determine whether MP modulated the expression of AQP4 following SCI. With immunohistochemical detection of AQP4, we found this water channel primordially expressed in the white matter-located glia limitans under baseline conditions. Variable degrees of AQP4 expression were found along the white matter, so we subdivided our analysis into 3 regions: internal, medial, and external (Figure 4), in order to make a fair comparison of the AQP4 expression. We found that SCI by itself induced an increased expression of AQP4 24 h after injury, especially in the internal and medial portions, as compared to sham-operated rats (Figure 4), and that treatment with MP diminished such increase to baseline levels in the medial and external portions (Figure 4).

## 4. Discussion

Using a clinically relevant model of acute SCI, we determined the effects of MP on key pathophysiological events at early time points, namely, spinal cord swelling, expression of AQP4, and permeability of the BSCB, all of them related to spinal cord edema, a major factor promoting secondary damage in neurotrauma. In spite of the proposed beneficial outcome of attenuating neuroinflammatory processes elicited by SCI, high doses of MP bear severe side effects that include a persistent state of immunosuppression that leads to infections and metabolic complications as well as pulmonary and adrenal insufficiency and gastrointestinal ulcers and bleeding [2, 3, 25] and even an increased risk of pneumonia and sepsis [26]. MP has been proven to reduce inflammation by blocking cytokine cascades and inhibiting T cell activation and extravasation, which are important mechanisms of neurodegeneration under chronic inflammatory conditions such as multiple sclerosis [27], and for many decades now, it has been thought that MP contributes to reducing neuroinflammation and to some extent relieving the complications of SCI associated with mechanisms of secondary damage such as BSCB alterations and edema formation. However, most of these presumed effects remain still to be determined. Overall, under our experimental conditions during the acute phase posttrauma, we did not find any improvement in motor performance following MP administration at the dose suggested by the NACIS II trial. Similar results have been previously reported [28].

The alterations found in the BSCB integrity were larger in the animals treated with MP, which correlated well with larger vasogenic edema. How MP induced such enhanced disruption of the spinal barrier needs to be further elucidated. In this sense, it has long been known that water accumulation occurs in the spinal cord following trauma and that the amount of edema correlates with the severity of the lesion [29–31]. Even more, it has been suggested that the extent of edema is proportionally associated with the level of motor dysfunction elicited by trauma [32]. Spinal edema that spans more than one vertebral segment is associated with greater deficits than edema with smaller areas [33], and a smaller edema size is related to better improvement in motor performance [34]. Also, there is evidence of increased cell swelling in the acute period following SCI [35]. Nonetheless, it has been proposed that spinal vasogenic edema generated through a leaky BSCB might not be as detrimental given that there is a circumferential expansion of the spinal cord with more space to expand as compared to that of the brain [36]. However, following SCI, the reduction of edema through the administration of a hypertonic saline solution improves spinal cord perfusion [37], suggesting that there is an increased pressure in the column at the injury site. In the same sense, it has been shown that spinal edema increases secondary tissue damage by the compressive effect increasing ischemia [38].

The early formation of edema following SCI has been widely documented before, and it has been established that it is mainly due to BSCB disturbances [39]. It has been reported that cytotoxic edema is decreased by the suppression of AQP4 but, on the contrary, the vasogenic type is worsened [40], suggesting that water clearance from the extracellular space is channeled through this protein. Accordingly, AQP4 deletion exacerbates vasogenic edema in the CNS, which has led to the suggestion that water enters CNS parenchyma through AQP4-independent mechanisms such as an impaired BSCB, but it is required for edema resolution [15, 41]. However, these mechanisms need to be further studied, since contradictory findings exist in which AQP4 deletion reduced spinal cord edema and improved neurological outcome in a compression model of a mouse spinal lesion [42].

We hypothesized here that MP could contribute to improving tissue healing and reducing inflammation by means of modulating AQP4 expression in the spinal cord area surrounding the primary lesion and in such a way contribute to reducing vasogenic edema and its associated consequences. Our findings fell quite in the opposite direction as we report a reduction in the astrocytic AQP4 expression following MP administration in injured animals, thus worsening edema. We centered our analyses on the glia limitans adjacent to the core of the lesion since in these cells, the expression of AQP4 is higher as compared to that in other regions of the spinal cord parenchyma, namely, the gray matter. At a very short time following the lesion (24 h), we did detect a significant increase in the expression of AQP4, especially in the internal and medial regions in which we arbitrarily divided our analysis. This is in accordance with the previous reports that indicate that AQP4 is upregulated several days after the lesion [24]. Nonetheless, this is a very important time point to study the possible effects of MP administration on water channel expression since its scheme of administration involves high doses administered at very short times following trauma. It is interesting that 24 h following MP administration, AQP4 expression in the glia limitans was notably reduced, promoting a negative impact on the endogenous mechanisms of tissue healing rather than contributing to an improved outcome. The decreased expression of AQP4 in the spinal cord correlated well with increased water content in the injured animals treated with MP as compared to rats administered with vehicle alone. However, the sole administration of MP did not promote an increase in water content by itself, which makes sense because in such scenario, there were no disturbances of the BSCB, which is the proposed mechanism for the formation of vasogenic edema in an AQP4-independent manner. Interestingly, a retrospective study comparing SCI between MP treated and nontreated patients did not find a statistically significant difference between groups in the development of spinal edema assessed by MRI [43], indicating that the clinical use of MP was not efficacious at reducing tissue swelling. These observations go well along the cellular and molecular findings reported here.

A limitation of the current study is that assessments were made at a single time point after injury and treatment. Further animal studies, designed to determine the temporal pattern of changes observed here, are warranted for a better understanding of the molecular effects of MP.

## 5. Conclusions

In this study, we found that the administration of high doses of MP right after traumatic SCI increases tissue swelling by a mechanism that involves the suppression of AQP4 upregulation in spinal astrocytes, potentiating the pathological hallmark of the contusion. This also contributed to enhancing BSCB alterations. While the controversy on the use of MP at high doses after SCI continues, our study provides scientific evidence of the harmful effects of this therapy on the spinal tissue and supports the current motion to discontinue its use in the clinical practice.
